# Mechanisms of Cinnamomi Cortex against Diabetes Mellitus Explored by Network Pharmacology Combined with Molecular Docking and Experimental Validation

**DOI:** 10.2174/0118715303300442240820075910

**Published:** 2024-09-23

**Authors:** Jianqin Yu, Zijun Song, Lusheng Wang, Hongyu Yang, Hui Fan

**Affiliations:** 1 College of Pharmaceutical Sciences, Zhejiang University, Hangzhou 310058, China;; 2 Department of Pharmacy, Eye Hospital, Wenzhou Medical University, Hangzhou, 310020, China;; 3 Department of Pharmacy, Hangzhou Third People’s Hospital, Hangzhou, 310009, China;; 4 Hangzhou Pharmacare Information Technology Co., Ltd, Hangzhou, 310013, China

**Keywords:** *Cinnamomi cortex* (CC), type 2 diabetes mellitus (T2DM), network pharmacology, inflammation, peroxisome proliferator-activated receptor γ (PPAR-γ), interleukin-6 (IL-6)

## Abstract

**Objective:**

*Cinnamomi cortex* (CC), a traditional Chinese herbal medicine, exhibits antidiabetic properties, yet the underlying mechanisms are not fully understood. Our study combined network pharmacology, molecular docking, and experimental validation to elucidate the antidiabetic mechanisms of CC.

**Methods:**

Active components of CC and their potential antidiabetic targets were identified through TCMSP, DisGeNET, and GeneCards. The PPI networks were constructed with STRING and analyzed with Cytoscape, while GO and KEGG analyses utilized the DAVID database. Molecular docking with core targets was performed using Autodock Vina. The efficacy of CC in diabetes mellitus was evaluated through H&E staining, qPCR, and Western blot in the T2DM mouse.

**Results:**

Eleven active components and sixty-six potential antidiabetic targets of CC were identified. The enrichment analysis revealed 288 GO terms and 37 pathways. The molecular docking showed high affinity for PPAR-γ and IL-6 receptors. *In vivo* studies further confirmed CC's ability to modulate PPAR-γ and IL-6, contributing to its antidiabetic effects.

**Conclusion:**

CC manages diabetes by regulating the PPAR-γ pathway and suppressing associated inflammation, providing a multi-pathway therapeutic approach.

## 1 INTRODUCTION

Diabetes mellitus, a chronic metabolic disorder, is divided into two primary types: Type 1 diabetes mellitus (T1DM), which is insulin-dependent, and Type 2 diabetes mellitus (T2DM), which is not insulin-dependent [1]. In patients with diabetes, inefficient glucose utilization occurs either due to inadequate insulin production or reduced insulin sensitivity, often resulting in elevated blood glucose levels. This hyperglycemic condition can lead to serious complications, including vascular diseases, retinopathy, nephropathy, neuropathy, and foot ulcers, significantly impacting various organ systems [2]. Despite the lack of a cure and the presence of numerous complications, diabetes remains a critical global health issue. The mainstay of clinical management for diabetes typically involves drugs such as metformin, sulfonylureas, thiazolidinediones, GLP-1 receptor agonists, DPP-4 inhibitors, and SGLT2 inhibitors [3]. However, these medications are associated with risks, including hypoglycemia and weight gain, and long-term high-dose usage may increase the risk of malignancies and cardiovascular diseases [4]. In addition, the physicochemical properties of these drugs pose challenges to their development. The study has proven, though, that protein sequence oscillations can aid in predicting protein structure and function, which might be of aid in drug development [5]. The peptides' general property is dependent on the protein sequence oscillation, and this may make the peptide structure unstable. Sequentially dependent are also the peptide activities, which may result in a low therapeutic window [6]. Consequently, these drawbacks may necessitate longer cycles to optimize peptide sequence design and stability.

Chinese herbal medicine, recognized as an alternative therapy for diabetes, has gained increasing attention. Its advantages lie in causing fewer side effects than synthetic drugs and effectively targeting multiple aspects of diabetes, including hyperglycemia, inflammation, and oxidative stress. This multifaceted approach aligns well with the growing demand for personalized medication among diabetic patients [7]. Consequently, delving into the therapeutic potential of Chinese herbs in treating diabetes holds substantial promise.

*Cinnamomi cortex* (CC), derived from the dried bark and branches of the *Cinnamomum* plant in the Lauraceae family, is predominantly harvested in China's Guangxi and Guangdong regions [8]. It is celebrated for its ability to warm the body's core, dispel cold, enhance blood circulation, and relieve pain [9]. CC is rich in various active components, which contribute to its significant anti-inflammatory, antibacterial, antioxidant, and antitumor properties [10]. The volatile oils in CC, such as coumarin, rhododendron, cinnamyl alcohol, and cinnamaldehyde, have potent antibacterial activity and can induce bacterial apoptosis by targeting the GyrA pathway to produce (ROS) [11, 12]. In addition, the non-volatile components of CC, such as terpenes, flavonoids, and polyphenolic compounds, act as antioxidants by scavenging excess oxygen free radicals and activating antioxidant enzymes [13]. Clinically, CC is widely utilized to treat conditions such as arthritis, abdominal pain and diarrhea, and dysmenorrhea [14]. Recent research has unveiled additional roles of CC in the regulation of glucose and lipid metabolism. Notably, daily CC intake has been linked with significant reductions in triglycerides (TG), total cholesterol (TC), and low-density lipoprotein (LDL) levels in diabetic patients [15]. Further investigations have illuminated that CC’s ability to modulate glucose and lipid metabolism is largely attributed to its impact on glucose transporter type 4 (GLUT4), thereby enhancing insulin sensitivity [16]. This attribute, coupled with CC's noted anti-inflammatory effects and its regulatory influence on glucose and lipid metabolism, underscores the importance of exploring its therapeutic applications in diabetes. However, despite a wealth of research supporting the therapeutic benefits of CC in diabetes management, the intricate composition of its active constituents and the diversity of its targets complicate the understanding of its exact mechanisms. The specific components within CC responsible for its antidiabetic effects remain to be conclusively identified.

Network pharmacology, integrating computer science and bioinformatics, is used to study drug mechanisms and disease pathways by analyzing interactions from the molecular to the organ level. Its ability to focus on multiple components and targets makes it particularly effective for understanding the intricate mechanisms of traditional Chinese herbal medicine [17]. Molecular docking is a crucial technique in the discovery of herbal medicines. Molecular docking identifies possible therapeutic components in natural plants by predicting the ligand-receptor protein binding affinity [18]. The use of molecular docking technology greatly reduces the time and cost associated with traditional experimental procedures and improves the efficiency of drug discovery [19]. In our study, we apply these approaches, complemented by experimental methods, to investigate the therapeutic effects and mechanisms of CC in treating diabetes. This combination of advanced computational techniques and rigorous experimental validation not only deepens our understanding of the antidiabetic effects of CC but also provides a powerful framework for elucidating the complex mechanisms underlying the antidiabetic effects of traditional Chinese medicines. This integrated approach paves the way for the development of more effective, targeted therapeutic strategies in the future.

## 2 MATERIALS AND METHODS

### 2.1 Materials

*Cinnamomi cortex* (CC) was sourced from Gansu Guofeng Medicine Co., Ltd. (Gansu, China). Streptozocin (STZ) was purchased from Beijing Solarbio Science & Technology Co., Ltd. (Beijing, China).

### 2.2 Construction of T2DM Mice

Healthy male C57BL/6 mice (5 in a group), aged seven weeks and weighing 18-22 g, were acquired from Gempharmatech Co., Ltd. (Jiangsu, China). They were housed in a controlled environment, maintained at 22 ± 1°C and 50-55% relative humidity, under a 12-hour light/dark cycle. Furthermore, to prepare the CC solution, the CC was finely ground into powder form and then dissolved in distilled water. The dosage for the mice was determined based on the clinical dosage for humans and it was adjusted according to the 'equivalent dose ratio table based on body surface area' between humans and animals [20]. Prior to molding, C57BL/6 mice were split into normal and T2DM groups. After 6 weeks on a high-fat diet (HFD), T2DM mice fasted for 12 hours and then received a 100 mg/kg STZ injection, while the normal group had a standard diet and saline injections. The success of T2DM induction was verified 7 days later by measuring fasting blood glucose (FBG) levels, with ≥11 mmol/L as the threshold. T2DM mice were then categorized into a model group, a low-dose CC group (CCL, 100 mg/kg), and a high-dose CC group (CCH, 600 mg/kg), receiving respective treatments for 4 weeks. The normal and model groups received equal volumes of water.

### 2.3 Screening of Active Compounds of CC

Active chemical ingredients of CC were obtained from the Traditional Chinese Medicine Systems Pharmacology (TCMSP, https://tcmspw.com/tcmsp.php), selecting potential active compounds with an oral bioavailability (OB) of ≥ 30% and drug-likeness (DL) of ≥ 0.18 as criteria.

### 2.4 Prediction of Compound Targets

The SMILES names of the active components of CC were obtained from PubChem (https://pubchem.ncbi.nlm.nih.gov), with their corresponding targets analyzed *via* Swiss Target Prediction (STP, http://www.swisstargetprediction.ch/).

### 2.5 Identification of Diabetes-related Targets

Diabetes-related disease genes were obtained from GeneCards (https://www.genecards.org/) and DisGeNET (https://www.disgenet.org). Furthermore, to analyze the relationship between CC and diabetes, an intersection was taken between the disease targets obtained and the potential action targets of the main compounds of CC, visualized by the VennDiagram package in R version 4.3.1.

### 2.6 Construction of the Drug-compound-target Network

Additionally, to elucidate the connections among active constituents of CC, their targets, and related diseases, the intersecting targets identified were mapped with the active compounds in CC using Cytoscape 3.9.0 (http://www.cytoscape.org/) to construct a visual “drug-compound-target” network.

### 2.7 Construction of the PPI Network

CC's potential diabetes targets were analyzed in STRING (https://string-db.org/). A protein–protein interaction (PPI) network was then visualized using Cytoscape 3.9.0, where CentiScape analyzed key topological metrics. The top 10 hub targets in the PPI network were identified using cytoHubba.

### 2.8 GO and KEGG Analysis

Gene ontology (GO) and Kyoto Encyclopedia of Genes and Genomes (KEGG) enrichment analyses were carried out *via* DAVID (https://david.ncifcrf.gov/) and visualized through the enrichplot and ggplot2 packages in R version 4.3.1.

### 2.9 Molecular Docking

In addition, to verify the effectiveness of the selected compounds and targets, molecular docking was performed between the active compounds and the top core targets. The commonly used clinical diabetes treatment drug, metformin, was used as a positive control [21]. Compound ligand files were sourced from PubChem and receptor structures from RCSB PDB (http://www.rcsb.org/). Native ligands of the proteins were extracted using PyMOL 2.3 (https://pymol.org/2/), and molecular docking was performed with Autodock Vina. The ligand with the best binding properties was then selected for visualization.

### 2.10 FBG and IPGTT

FBG and intraperitoneal glucose tolerance test (IPGTT) are key methods for diagnosing diabetes in mice. After 4 weeks of treatment, mice were fasted for 12 hours (water allowed) and then administered a 2 g/kg dose of 10% glucose intraperitoneally. Blood glucose was measured at 0, 30, 60, 90, and 120 minutes post-injection, with the initial measurement indicating each mouse's FBG level.

### 2.11 H&E Staining

The liver, pancreas, and white adipose tissues of mice were preserved for 24 hours in a 4% formaldehyde solution, embedded in paraffin, sectioned into 5-6 μm paraffin slices, and stained with hematoxylin-eosin (H&E) (Solarbio, Beijing, China). The sections were then mounted with neutral gum media. The examination of the specimens was carried out with a Leica DM3000 microscope (Wetzlar, Germany) to ascertain any histopathological alterations.

### 2.12 Insulin Assay

Orbital venous blood was obtained from mice and left to clot naturally at room temperature, followed by centrifugation at 3000 × *g* for 15 minutes to separate the serum. The fasting insulin level in mouse serum was determined using an Insulin Assay Kit (Nanjing Jiancheng Bioengineering Institute, Nanjing, China).

### 2.13 MDA Assay

Malondialdehyde (MDA), a product of lipid peroxidation, reflects the level of oxidative stress in diabetes. The liver tissue was ground and homogenized with saline to form a 10% homogenate. The supernatant was obtained by centrifugation at 12,000 × *g*. Subsequently, according to the instructions of the MDA Assay Kit (Beyotime, Shanghai, China), samples were heated in a boiling water bath for 15 minutes, and the absorbance was measured at 532 nm.

### 2.14 SOD Assay

Superoxide Dismutase (SOD) is an enzyme responsible for the elimination of superoxide radicals (O^2-^), protecting cells from free radical-associated damage. The liver tissue was ground and homogenized with saline to form a 10% homogenate. The supernatant was obtained by centrifugation at 12,000 × g. Subsequently, following the Total Superoxide Dismutase Assay Kit with WST-8 (Beyotime, Shanghai, China) instructions, the samples were incubated at 37°C for 30 minutes, and the absorbance was measured at 450 nm.

### 2.15 Quantitative PCR

The liver RNA was isolated using Trizol^®^ Reagent (Thermo Fisher Scientific, Waltham, MA, United States) and reverse transcribed into cDNA with the PrimeScript™ kit (Takara, Dalian, China). The qPCR amplification of cDNA and specific primers was performed using SYBR™ Green (Thermo Fisher Scientific, Waltham, MA, United States). The target gene expression levels were normalized to the endogenous control gene *β-Actin*, and relative gene expression was calculated using the 2^−ΔΔCt^ method. Below are the primer sequences: *PPAR-γ*: f: 5'-GGAAGACCACTCGC ATTCCTT-3', r: 5'-GTAATCAGCAACCATTGGGTCA-3'; *IL-6*: f: 5'-TCCTCTCTGCAAGAGACTTCCATC-3', r: 5'-TGGTTGTCACCAGCATCAGTCC-3'; *INSR*: f: 5'-ATGG GCTTCGGGAGAGGA-3', r: 5'-GGATGTCCATACCAG GGCAC-3'; *IRS-1*: f: 5'-CAAGACGCTCCAGTGAGGAT-3', r: 5'-TCATTCTGCTGTGATGTCCA-3'; *PGC1-α*: f: 5'-CCGTAAATCTGCGGGATGATGGAG-3', r: 5'-TCAAGA GCAGCGAAAGCGTCAC-3'; *GCK*: f: 5'-AGGCACGA AGACATAGACAAGGG-3', r: 5'-ACCATTGCCACCAC ATCCATCTC-3'; *G6PC*: f: 5'-ACTGTGGGCATCAATC TCCTCTG-3', r: 5'-GGCGTTGTCCAAACAGAATCCAC-3'; *CPT*: f: 5'-TGCAGCTCGCACATTACAAGGAC-3', r: 5'-CGAGTCATGGAAGCCTCATACGTG-3'.

### 2.16 Western Blot

Mouse liver tissue was ground in liquid nitrogen until powdered and lysed with protease, and the supernatant was removed by centrifugation at 4°C, 12,000 × *g*. The supernatant was then separated on a 12% SDS-PAGE gel. After that, the protein bands were transferred to a polyvinylidene fluoride (PVDF) membrane and then blocked with nonfat powdered milk. The samples were then incubated with anti-PPARγ, anti-GLUT1, and anti-GLUT4 antibodies (Proteintech, Chicago, IL, United States) overnight. After incubation, the membrane was incubated with the secondary antibody (Proteintech, Chicago, IL, United States). Finally, chemiluminescence detection was performed using substrates (Pierce, Rockford, IL, United States).

### 2.17 Statistical Analysis

GraphPad Prism 6.0 (GraphPad, San Diego, CA, United States) was used for all statistical analyses. A comparison of multi-group data was carried out with a one-way analysis of variance (ANOVA) followed by Dunnett’s test. “#” and “##” indicate significant differences from the normal group at the *p* < 0.05 and *p* < 0.01 levels, respectively. “*” and “**” indicate significant differences from the model group at the *p* < 0.05 and *p* < 0.01 levels, respectively.

## 3 RESULTS

### 3.1 Exploring the Effects of CC on T2DM Mice

**Fig. (1):**
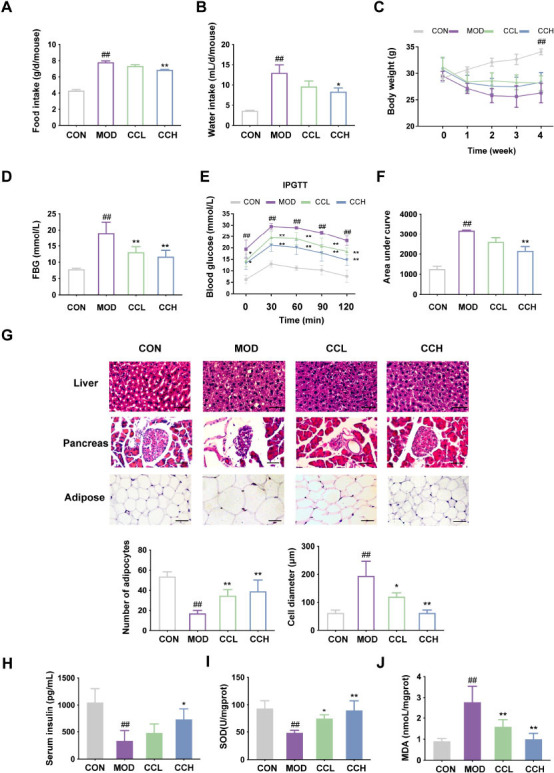
CC mitigates the diabetes symptoms in T2DM mice induced by HFD/STZ. **A**) Food intake; **B**) Water intake; **C**) Body weight; **D**) FBG; **E**) IPGTT; and **F**) AUC; **G**) HE staining of liver, pancreas, and white adipose tissue sections; the graph below represents the count and diameter of adipocytes; **H**) Detection of insulin concentration in serum; **I**) Detection of SOD enzyme activity in liver tissue; **J**) Detection of MDA content in liver tissues. Data are expressed as mean ± SD (n=5).

Diabetes typically leads to increased food and water intake and weight loss. In our study, diabetic mice (model group) consumed more food and water than normal mice. However, CC-treated mice (CCH group) showed significant improvement in these aspects (Figs. **1A** and **B**). While normal mice gained weight over time, the model group exhibited weight loss, with the CC group showing a slight decrease (Fig. **1C**). Fasting blood glucose (FBG), a key diabetes diagnostic, was notably higher in the model group than in normal mice. However, FBG levels significantly dropped in both CCL and CCH groups, indicating dose-dependency (Fig. **1D**). Additionally, IPGTT and area under the curve (AUC) results further revealed that CC moderated the abnormal blood glucose spike post-glucose intake, enhancing glucose tolerance in diabetic mice (Figs. **1E** and **F**).

The development of diabetes typically coincides with fatty pathological alterations in tissues. H&E staining results showed white lipid droplets filling the liver cells of the model group, with adipocyte volume abnormally larger than that of the normal group. Additionally, islets in the pancreas of diabetic mice underwent atrophy and were markedly irregular in shape. In the CC groups, a decrease in lipid droplets within liver cells and improvement in adipocyte enlargement were observed, and islet morphology was normalized in CC-treated mice compared to diabetic mice. These results indicate that CC is effective in alleviating tissue lipid accumulation and islet cell apoptosis caused by diabetes mellitus (Fig. **1G**). Notably, insulin levels in the serum of CC-treated mice were significantly elevated, suggesting that CC may have a pancreatic β-cell protective effect (Fig. **1H**).

Oxidative stress contributes to the worsening of diabetes and its complications through lipid peroxidation. The SOD and MDA assays revealed that the model group mice exhibited significantly reduced SOD activity and elevated MDA levels compared to the normal group, indicating pronounced oxidative stress damage in the diabetic mice. Following treatment with CC, the levels of SOD were significantly increased compared to the model group, and the MDA content was significantly decreased, indicating that CC can modulate the oxidative stress imbalance induced by HFD/STZ and provide a protective effect (Figs. **1I** and **J**).

### 3.2 Construction of Drug-compound-target and PPI Networks

**Fig. (2):**
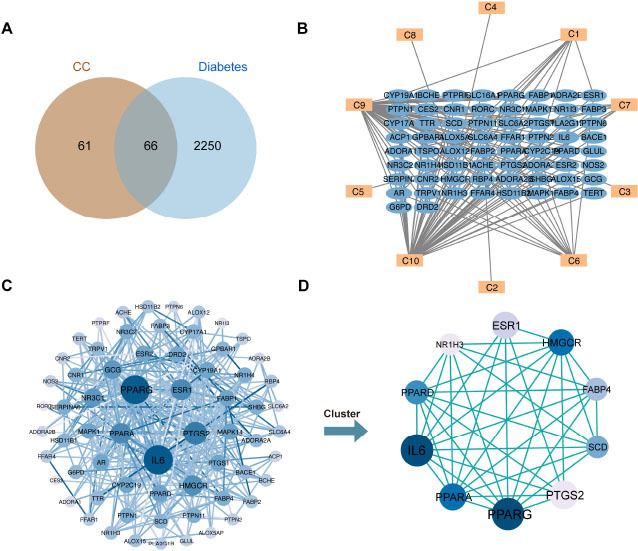
Study of the interaction targets. **A**) Identification of therapeutic targets for CC against diabetes mellitus; **B**) Drug-component-target interaction network of CC targets against diabetes mellitus, with brown representing compounds of CC and blue representing targets, respectively; **C**) PPI network of 66 targets of CC against diabetes mellitus; **D**) PPI sub-network of the top 10 hub targets of CC against diabetes mellitus, where the volume and color of the nodes reflect the degree of connectivity (the larger volumes and bluer color indicate a higher degree).

Furthermore, to elucidate the anti-diabetic mechanisms of CC, a network pharmacology approach was employed to predict its active ingredients and their corresponding targets. The research identified 11 active compounds (Table **1**). Utilizing GeneCards and DisGeNET, a total of 2316 diabetes-related target genes were discerned. A cross-analysis between the potential targets of CC and diabetic genes identified 66 probable anti-diabetic targets of CC (Fig. **2A**). Moreover, the drug-compound-target network relating to active compounds and the disease was established with Cytoscape (Fig. **2B**).

A PPI network was constructed through further analysis with STRING. This network includes a total of 66 nodes and 334 edges, featuring an average node degree of 10.1 and an average local clustering coefficient of 0.508. The topological analysis revealed proteins with high connectivity, such as IL-6, peroxisome proliferator-activated receptor γ (PPAR-γ), prostaglandin-endoperoxide synthase (PTGS2), estrogen receptor 1 (ESR1), PPAR-α, among others (Fig. **2C**). A sub-network comprising the 10 most relevant core targets was identified through advanced clustering analysis of the PPI network, with IL-6 and PPAR-γ occupying predominant positions, suggesting their central roles in CC's mediation of anti-diabetic effects (Fig. **2D**).

### 3.3 Enrichment Analysis of GO and KEGG

The enrichment analysis of 66 potential anti-diabetic targets of CC conducted through the DAVID database identified 189 biological processes (BP), 31 cellular components (CC), and 68 molecular functions (MF). The top 10 entries for each section are shown in Fig. (**3A**). The results indicate that CC modulates diabetes through various biological functions, including regulating the RNA polymerase II transcription process, intracellular receptor signaling pathways, glucose homeostasis, and inhibiting inflammatory responses. The KEGG enrichment analysis identified 37 related signaling pathways, with the top 20 significant pathways shown in Fig. (**3B**). Notably, the PPAR signaling pathway plays a key role in regulating lipid metabolism, inflammation, and insulin sensitivity. The activation of PPAR helps improve insulin sensitivity, reduce insulin resistance, and inhibit inflammation mediated by inflammatory factors. Combined with previous results, this suggests that CC may exert its anti-diabetic effects by activating the PPAR signaling pathway and inhibiting IL-6-mediated inflammation.

### 3.4 Molecular Docking

The PPI network and enrichment results indicated that CC's anti-diabetic activity is associated with PPAR signaling pathways and inflammatory responses. The molecular docking of 11 active components of CC with PPAR-γ and IL-6 proteins was performed using metformin as a positive control. Table **2** presents the binding energy scores. Binding energy less than -7 kJ/mol signifies better binding activity, with lower energies indicating better docking effectiveness. It is shown that all active components exhibit binding affinities with PPAR-γ greater than metformin (-4.9 kJ/mol), and most of them demonstrate binding energy lower than -7 kJ/mol, suggesting a higher binding stability of CC with PPAR-γ. In addition, the binding affinities of active components with IL-6 are also better than metformin (-4.5 kJ/mol). These findings imply that CC’s active components are capable of stably binding with PPAR-γ and IL-6, which may be crucial for their anti-diabetic effects. Fig. (**4**) displays the docking results for the top 1 target.

### 3.5 Study on the Anti-diabetic Mechanism of CC in T2DM Mice

**Fig. (5):**
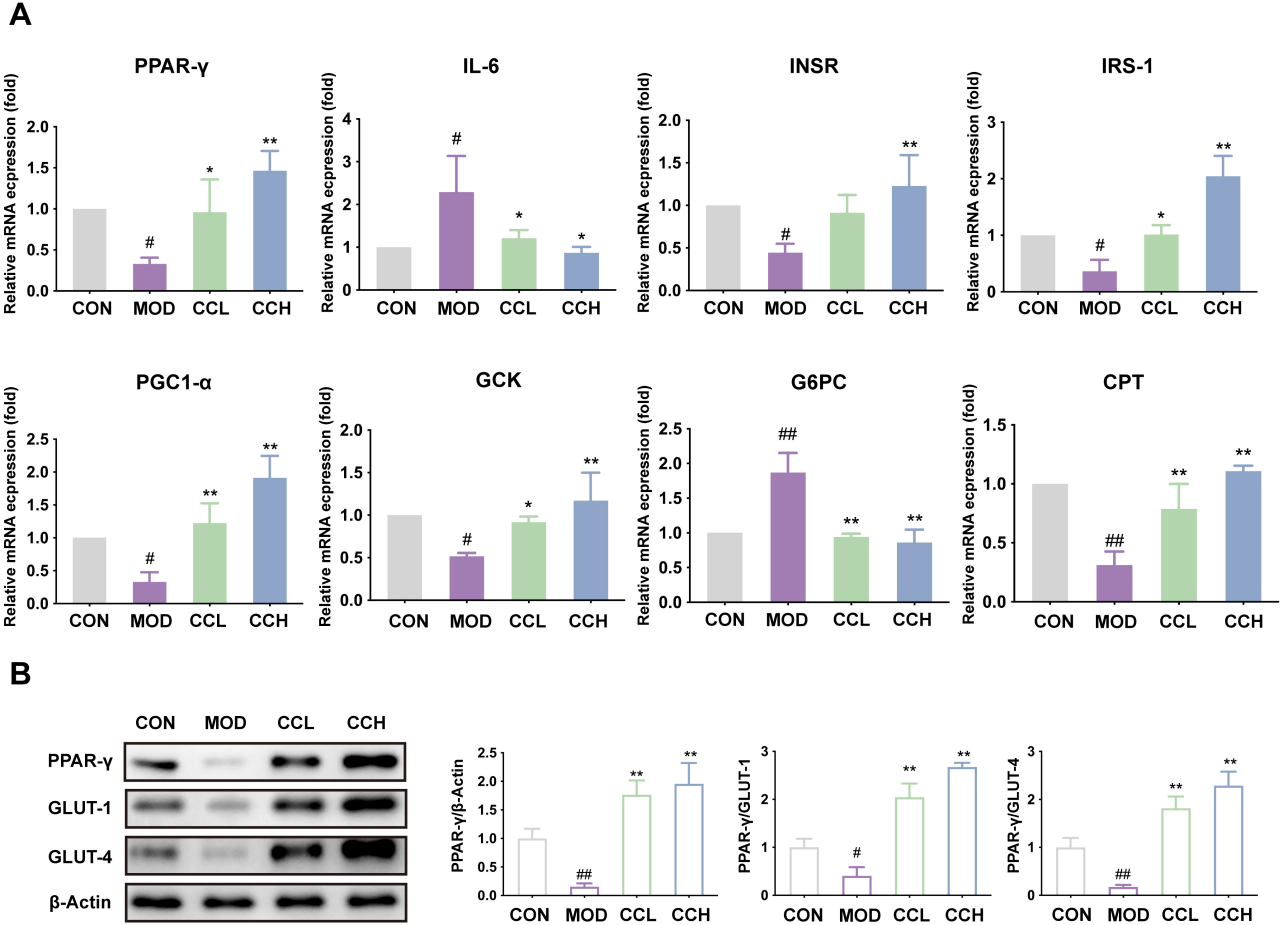
**A**) Changes in the mRNA levels of diabetes-related genes in T2DM mice by qPCR. **B**) Changes in the protein levels of PPAR-γ, GLUT-1, and GLUT-4 in T2DM mice by western blot. Data are expressed as mean ± SD (n=3).

Network pharmacology and molecular docking studies have identified PPAR-γ and IL-6 as the core targets of CC in the treatment of diabetes. PPAR-γ plays a crucial regulatory role in glucose metabolism, fatty acid metabolism, and insulin signaling transduction by acting on downstream target genes. The qPCR results showed a significant reduction in *PPAR-γ* mRNA levels in T2DM mice. Additionally, the mRNA levels of insulin receptor (I*NSR*), insulin receptor substrate 1 (*IRS1*), peroxisome proliferator-activated receptor γ coactivator 1-α (*PGC1-α*), glucokinase (*GCK*), and carnitine palmitoyl transferase (*CPT*) in diabetic mice were lower than in normal mice, while glucose-6-phosphatase catalytic (*G6PC*) mRNA levels were significantly higher, indicating severe glucose and lipid metabolism disorders and insulin signaling transduction damage in diabetic mice. As downstream targets of PPAR-γ regulation, these results indicate compromised PPAR-γ signal transduction in diabetic mice. In contrast, CC significantly reversed the aberrant expression of these diabetes-related genes induced by HFD/STZ. Moreover, CC treatment significantly reduced the *IL-6* levels, preventing the progression of diabetes (Fig. **5A**).

In addition, the results of the western blot showed that PPAR-γ and GLUT1, GLUT4 protein levels were significantly reduced in T2DM mice, suggesting severe impairment of glucose metabolic function. In contrast, CC treatment significantly reversed the abnormal expression of GLUT1 and GLUT4 proteins and promoted PPAR-γ protein expression (Fig. **5B**). Overall, CC likely exerts its therapeutic effects on diabetes by modulating the PPAR-γ mediated signaling pathways and inhibiting inflammation, thereby enhancing insulin sensitivity, and regulating glucose and lipid metabolism.

## 4 DISCUSSION

CC, a traditional Chinese medicinal herb, is known for its diverse properties, such as anti-inflammatory, antioxidant, and blood circulation enhancement. Our study revealed that administering aqueous extracts of CC to T2DM mice effectively reduced the elevated blood glucose levels caused by HFD/STZ and improved fatty lesions in the liver, pancreas, and adipose tissue. It is worth noting that CC extracts significantly ameliorated oxidative stress in mouse liver, suggesting that CC extracts are likely to circulate through the bloodstream to achieve this effect. Previous studies have highlighted the role of polyphenols and flavonoids in alleviating joint swelling and inflammation due to their potent antioxidant capabilities [14]. These compounds scavenge free radicals and chelate metal ions, thereby preventing oxidative damage to cells and tissues [22]. The accumulation of iron ions in the blood, particularly the ferrous ions, can cause overproduction of ROS in the body and result in lipids peroxidation within the cells. Finally, it ends up causing cell death, also known as iron death. These polyphenols can bind with iron ions, forming complexes in the body that do not allow the ion to be in a free state in the body [23]. Furthermore, it was reported that iron can be exported from enterocytes, hepatocytes, and macrophages to the bloodstream through iron transport protein-1 (FPN1). In contrast, polyphenols may further decrease the damaging effects of iron on oxidative damage by modulating the iron metabolic pathways [24]. When CC extracts enter the bloodstream, the polyphenols and flavonoids circulate throughout the body, exerting their antioxidant effects at various sites. These compounds can directly neutralize ROS and reactive nitrogen species (RNS), reducing oxidative stress levels in tissues.

Our study suggests that CC extracts are likely to ameliorate diabetes by reducing vascular endothelial cell dysfunction and inflammation and reducing tissue oxidative stress levels. Despite these findings, the underlying mechanisms of CC for the treatment of diabetes are still not fully understood. Therefore, we investigated the mechanism of action of CC in depth using a network pharmacology approach.

An analysis of the PPI network indicates that CC's anti-diabetic targets comprise IL-6, PPAR-γ, PTGS2, ESR1, PPAR-α, and others. IL-6 is significantly increased during the onset of diabetes, a condition often characterized by enhanced inflammatory activity [25]. In comparison to healthy individuals, individuals with prediabetes exhibit markedly higher serum levels of inflammatory cytokines IL-6 and TNF-α, indicating a possible connection between IL-6 and the development of T2DM [26, 27]. PTGS2, acting as the rate-limiting enzyme in prostaglandin synthesis from arachidonic acid, contributes to inflammation and pain transmission. Its overexpression can exacerbate glomerular injury in patients with diabetic nephropathy [28]. ESR1, a transcription factor, regulates the SLC2A4 gene, influencing various cellular signals. This modulation affects GLUT4, thus impacting blood glucose regulation [29]. PPARs, as ligand-activated nuclear receptors, interact with PPAR response elements (PPREs) and retinoid X receptors (RXRs) to regulate gene transcription. Their subtypes, PPAR-α, PPAR-β/δ, and PPAR-γ, have distinct functions: PPAR-α controls fat metabolism in fasting, PPAR-γ improves insulin sensitivity *via* fat management, and PPAR-β/δ enhances mitochondrial activity for glucose and fatty acid metabolism [30, 31]. Drugs like the fibrate fenofibrate and TZD class drugs pioglitazone and rosiglitazone, which target PPAR-α and PPAR-γ, are commonly employed in diabetes treatment [32]. Combined with our research, this implies that CC could have antidiabetic properties by influencing glycolipid metabolism and inflammatory response targets.

The enrichment analysis suggests that CC's antidiabetic effects are linked to maintaining glucose homeostasis, reducing inflammatory responses, and regulating intracellular receptor pathways. Diabetes progression often leads to abnormal insulin secretion, disrupting glucose homeostasis and causing high blood glucose levels. Elevated blood glucose levels can trigger excessive ROS production in mitochondria, resulting in oxidative stress and various diabetic complications [33]. Excess ROS, resulting from disrupted glucose metabolism, can trigger chronic inflammation by activating the transcription factor NF-*κ*B [34]. Signaling pathways are vital in these processes, significantly influencing the development of T2DM. Key pathways like PI3K/AKT, AMPK, MAPK, WNT, and PPARs are involved in regulating insulin effects and glucose metabolism, which is crucial for managing diabetes progression [35]. Consistent with the results of enrichment analysis, pathway analysis further indicates that the PPAR signaling pathway might play a central role in mediating CC's antidiabetic effects. Building upon these findings, our molecular docking results confirmed that various active components of CC effectively bind with PPAR-γ and IL-6, exhibiting substantial binding stability.

PPAR-γ is closely linked to diabetes due to its capacity to regulate genes involved in fatty acid oxidation and notably reduce oxidative stress caused by inflammatory factors [36, 37]. *In vivo* validation results showed reduced PPAR-γ expression in T2DM mice and abnormal expression of downstream target genes, indicating impaired PPAR-γ signaling pathway activity. INSR and IRS-1, target genes of PPAR-γ, play critical roles in insulin signaling, with their expression levels directly influencing the body’s insulin sensitivity [38, 39]. PGC1-α, when dysregulated, indicates mitochondrial dysfunction, which results in excessive ROS production and subsequent oxidative stress damage [40]. GCK serves as the rate-limiting enzyme in glycolysis, transforming glucose into G6P. Conversely, G6PC dephosphorylates G6P, aiding in gluconeogenesis. Both enzymes are essential for maintaining glucose homeostasis [41, 42]. CPT, the key enzyme in fatty acid oxidation, facilitates β-oxidation of fatty acids. Its diminished expression can lead to mitochondrial dysfunction [43]. CC has shown efficacy in reversing the abnormal expression of PPAR-γ target genes caused by HFD/STZ, restoring the balance of glucose and lipid metabolism, and boosting PPAR-γ expression, indicating its potential antidiabetic effects *via* the PPAR-γ pathway.

PPAR-γ activation enhances the transcription of genes involved in glucose uptake and utilization, such as GLUT4, which increases glucose uptake by adipose and muscle tissues, thereby lowering blood glucose levels [44]. Additionally, PPAR-γ upregulates the expression of GCK, promoting efficient glucose metabolism and reducing hyperglycemia. PPAR-γ also promotes the differentiation of preadipocytes into mature adipocytes, reducing circulating free fatty acids and triglycerides, leading to more efficient fat storage, reduced lipotoxicity, and improved insulin sensitivity [45]. Furthermore, PPAR-γ activation reduces oxidative stress by increasing the expression of antioxidant enzymes like SOD and catalase, protecting cells from oxidative damage [46]. Chronic inflammation, a hallmark of T2DM, is alleviated by PPAR-γ through antagonizing the NF-*κ*B signaling pathway and inhibiting pro-inflammatory cytokines such as TNF-α, IL-6, and MCP-1 [47]. These mechanisms suggest that CC exerts multiple antidiabetic effects, including regulation of glucose and lipid metabolism, as well as antioxidant and anti-inflammatory effects, through PPAR-γ activation. Further research is needed to fully elucidate these pathways and confirm the therapeutic potential of CC in managing T2DM.

## CONCLUSION

CC could exert a therapeutic effect on T2DM by regulating the PPAR-γ signaling pathway. Our studies have clarified the mechanism of action of CC's antidiabetic effects, demonstrating its ability to enhance glucose metabolism, reduce oxidative stress, and alleviate inflammation. These findings provide a clear understanding of the efficacy and mechanism of action for CC's active ingredients, positioning them as promising candidates for the development of natural antidiabetic drugs and helping to avoid potential side effects associated with synthetic chemical drugs in the clinical treatment of diabetes, offering a safer and more effective therapeutic option.

## Figures and Tables

**Fig. (3):**
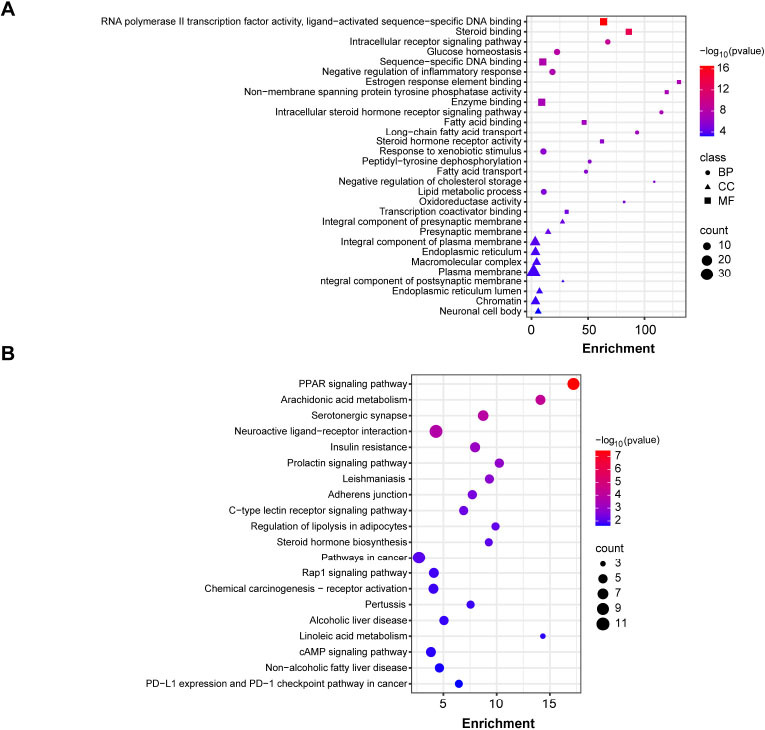
Enrichment analysis of intersection targets. **A**) The top 10 results of GO enrichment analyses for 66 overlapping targets; **B**) The top 20 results of KEGG enrichment analyses for 66 overlapping targets. The color represents the different *p*-values, and the size of the circle represents the counts.

**Fig. (4):**
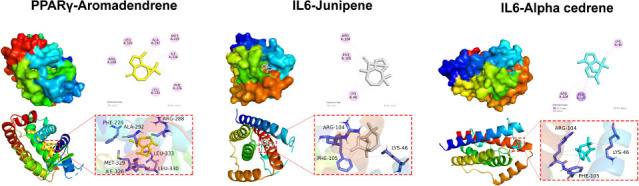
Visualization of top 1 binding energy for PPAR-γ and IL-6 with compounds of CC.

**Table 1:** Screened compounds of CC and their properties.

**No**	**Name**	**OB%**	**DL**
C1	Linoleic acid	41.90	0.14
C2	Copaene	29.47	0.12
C3	(+)-Aromadendrene	55.74	0.10
C4	Beta-Cubebene	32.81	0.11
C5	Junipene	44.07	0.11
C6	(+)-Sativene	37.41	0.10
C7	(+)-Ledene	51.84	0.10
C8	(-)-Caryophyllene oxide	32.67	0.13
C9	Diisobutyl phthalate	49.63	0.13
C10	(-)-Alpha-cedrene	55.56	0.10
C11	Oleic acid	33.13	0.14

**Table 2:** The binding energy for PPAR-γ and IL-6 with compounds of CC.

**No**	**Name**	**Target**	**Binding Energy (kJ/mol)**
Positive control	Metformin	PPAR-γ IL-6	-4.9 -4.5
C1	Linoleic acid	PPAR-γ IL-6	-6.1 -4.6
C2	Copaene	PPAR-γ IL-6	-7.3 -5.9
C3	(+)-Aromadendrene	PPAR-γ IL-6	-8 -6
C4	Beta-Cubebene	PPAR-γ IL-6	-7.3 -5.8
C5	Junipene	PPAR-γ IL-6	-7.7 -6.2
C6	(+)-Sativene	PPAR-γ IL-6	-7 -6
C7	(+)-Ledene	PPAR-γ IL-6	-7.3 -5.9
C8	(-)-Caryophyllene oxide	PPAR-γ IL-6	-7.7 -6
C9	Diisobutyl phthalate	PPAR-γ IL-6	-7 -5.3
C10	(-)-Alpha-cedrene	PPAR-γ IL-6	-7.5 -6.2
C11	Oleic acid	PPAR-γ IL-6	-5.9 -4.8

## Data Availability

All the data and supportive information are provided within the article.

## LIST OF ABBREVIATIONS

AUC: Area Under the Curve
BP: Biological Processes
CC: *Cinnamomi Cortex*
DL: Drug-likeness
FBG: Fasting Blood Glucose
GLUT4: Glucose Transporter Type 4
H&E: Hematoxylin-eosin
IL-6: Interleukin-6
IPGTT: Intraperitoneal Glucose Tolerance Test
KEGG: Kyoto Encyclopedia of Genes and Genomes
LDL: Low-density Lipoprotein
MDA: Malondialdehyde
OB: Oral Bioavailability
PPAR-γ: Peroxisome Proliferator-activated Receptor γ
PPI: Protein–protein Interaction
SOD: Superoxide Dismutase
T1DM: Type 1 Diabetes Mellitus
T2DM: Type 2 diabetes Mellitus
TC: Total Cholesterol
TG: Triglycerides
MF: Molecular Function
GO: Gene Ontology
